# Functional Diversity and Emerging Roles of Human NME/NDPK Group II Proteins

**DOI:** 10.3390/ijms27114871

**Published:** 2026-05-28

**Authors:** Bastien Proust, Helena Ćetković, Maja Jazvinšćak Jembrek, Maja Šutić, Lea Vrbančić, Maja Herak Bosnar

**Affiliations:** 1 Division of Molecular Biology, Ruđer Bošković Institute, Bijenička Cesta 54, 10002 Zagreb, Croatia; bproust@irb.hr (B.P.); cetkovic@irb.hr (H.Ć.); 2 Division of Molecular Medicine, Ruđer Bošković Institute, 10002 Zagreb, Croatia; jazvin@irb.hr (M.J.J.); msutic@irb.hr (M.Š.); lvrbanc@irb.hr (L.V.); 3 Faculty of Science, University of Zagreb, Horvatovac 102, 10002 Zagreb, Croatia

**Keywords:** NME, Nm23, nucleoside diphosphate kinase

## Abstract

NME/NDP kinases are enzymes primarily responsible for maintaining nucleotide balance in the cell. They arose early in evolution, during which they acquired additional biochemical and biological functions such as protein histidine kinase activity, DNA transcription and repair, and the binding and transfer of phospholipids. The human NME/NDPK family comprises 10 proteins divided into two groups. The well-documented Group I (NME1-4) is characterized by high sequence homology and a single active NDPK domain. In contrast, the remaining NME genes/proteins belong to the poorly understood and more heterogeneous Group II. They possess one or more NDPK domains and are divergent in their amino acid sequences. Except for NME6, they have been considered enzymatically inactive. In recent years, NME Group II proteins have attracted more interest from researchers, and new emerging evidence may change the established perspective.

## 1. Introduction

The *NME* gene/protein family, also known as the *NM23/Awd* family, encodes nucleoside diphosphate kinases (NDPKs), enzymes that catalyze the transfer of the terminal phosphate from nucleoside triphosphates to diphosphates through a high-energy phosphohistidine intermediate [1]. Nucleoside diphosphate kinases are characterized by their NDPK domain, a specific protein sequence/structure that forms the center for NDPK activity. The NDPKs catalyze the reaction through a ping-pong mechanism: first ATP enters the NDPK catalytic site and its γ-phosphate group is transferred to the main catalytic histidine (H118 in human NME1). The NDPK is, thus, in a transient, high-energy phosphohistidine state. ADP is released, and another NDP enters the catalytic site. The phosphate group is then transferred to the NDP as a γ-phosphate group, forming NTP [2]. In addition to maintaining the general cellular NTP pool, some NME proteins appear to provide a local supply of NTPs such as GTP to fuel GTPases [3]. Overall, the NME/NM23 family represents a multifunctional group of proteins whose activities extend far beyond nucleotide metabolism, influencing processes essential for cell survival, differentiation, and disease progression [4].

Members of this family are evolutionarily conserved and found in nearly all living beings (except mycoplasmas), confirming their fundamental role in cellular physiology [5]. In humans, the family includes several paralogs (NME1–NME9) each with distinct but also overlapping functions in the cell [1]. NME1 was originally identified because its expression inversely correlated with metastatic potential in melanoma cells [6]. Subsequent studies confirmed that reduced NME1 expression is associated with increased metastatic behavior in several tumor types [7]. This discovery positioned *NME1* as a founder of a novel group of gene-metastasis suppressors [7]. Beyond cancer biology, NME proteins participate in a variety of cellular processes such as vesicular trafficking, signal transduction, cytoskeleton regulation, and ciliary function [8,9]. NME proteins are typically localized in the cytoplasm but can also be found in the nucleus, mitochondria, and extracellular space depending on the paralog [1,10,11,12].

Several features of NME proteins are important for oligomer assembly and enzymatic activity, with the Kpn loop in the NDPK domain and the C-terminal extension being the most studied. The Kpn loop stabilizes NME complexes at the trimer interface during hexamer formation. The C-terminal region is highly variable in sequence among different NME proteins and has been shown to be important for hexamer oligomerizations. A Kpn mutation combined with a C-terminal truncation is often associated with oligomer dissociation and loss of enzymatic activity [13,14,15].

NME proteins can form homohexamers or heterohexamers as reported in eukaryotes, archaea, and some bacteria [16,17,18,19,20]. In other bacteria, NMEs have been reported to oligomerize as tetramers [21,22,23]. The structures, folding, and stability of NME proteins from a wide range of species were reviewed by Georgescauld and coworkers [24]. It is generally accepted that monomers first assemble as head-to-tail dimers before aggregating into tetramers or hexamers. Until recently, it was widely accepted—almost as a dogma—that monomeric NME proteins cannot be enzymatically active. However, recent investigations, which will be discussed later in this paper, suggest that their enzymatic activity could be driven differently, perhaps through interaction with another protein.

**The Group II NME/NDPK proteins**

In vertebrates, 10 members of the NME/NDPK family have been recorded to date. They are divided into two groups: NME1–NME4 and NME5–NME9, based on phylogenetic studies and exon/intron structure. NME10 protein has passed through a separate evolutionary history since it seems evident that its NDPK domain was inserted relatively recently and therefore, cannot be classified in either of the two groups. The well-studied Group I members (*NME1-4*) appear to have emerged from a common ancestral gene around the time of teleost radiation [25]. They show a high degree of mutual homology and are also homologous to orthologs across species. Their enzymatic activity is well-documented. *NME1* and *NME2* encode cytosolic NDPK hexameric isoenzymes and account for 80% of the NDPK activity in the cell [26]. NME3 and NME4 are mitochondrial NDPKs that form homohexamers and are also linked to human diseases [27,28,29]. To date, as many as three NMEs are targeted to mitochondria: NME3 localizes to the mitochondrial outer membrane, NME4 localizes to the mitochondrial inner membrane, facing either the intermembrane space or the matrix, and NME6, a member of the Group II, is localized mostly in the matrix space [27,30,31].

In contrast to Group I, the members of the NME Group II (NME5–NME9) are more divergent among themselves (28–45% identity), and are dissimilar to Group I proteins (25–34% identity) [32]. Generally, it has been considered that they do not possess NDP kinase activity [33]. Their oligomeric state has not yet been elucidated. *NME5–NME8* were already present in the genome of the common ancestor of choanoflagellates and metazoans and emerged around the time of eukaryote radiation. Most Group II genes and proteins are present in early-branching eukaryotic lineages. Evolutionary studies of the Group II genes/proteins underscore their necessity in the physiology of every living cell, although there has been no systematic research to reveal their function [5,25]. In recent years, more detailed and systematic investigations have been undertaken on NME Group II members. Some of these studies, especially those on NME6, have revealed previously unrecognized characteristics of this protein subfamily. In this paper, we provide an overview of the most recent research on Group II NME proteins and attempt to interpret these findings from a renewed scientific perspective.

**NME5**

The human *NME5* gene (also known as *nm23-H5*, *NM23H5*, *NDPk5*, *RSPH23*) is located on chromosome 5 at cytogenetic band 5q31.2 in the human genome. *NME5* is reported to be most abundantly expressed in human testicular germ cells, distinguishing it from other NME family members. It has been shown to localize in the axonemal microtubules of spermatids and sperm flagella, indicating a possible role in regulating sperm motility [34,35]. In mice, *NME5* is specifically expressed during spermatogenesis and spermiogenesis, suggesting a role in male germ cell development [36]. Furthermore, the antioxidant enzyme glutathione peroxidase 5 (GPX5) is regulated by NME5 in mice at both the expression and activity levels. These results show that *NME5* expression plays a critical role in spermiogenesis by increasing the cellular GPX5 levels to eliminate reactive oxygen species (ROS). The authors suggest that NME5 is part of an antioxidant protective pathway in the testis, helping to control ROS levels and support normal sperm maturation through the regulation of GPX5 [37]. Further studies showed that NME5, together with Hk1, Akap4, Arih1, Rassf7 and Tubb4b, is strictly associated with active spermatogenesis in both mice and lizards [38]. A novel homozygous frameshift variant in NME5 was recently identified in an infertile man diagnosed with acephalic spermatozoa syndrome (ASS). The study provides the first direct genetic and clinical evidence that *NME5* is a causative gene for human ASS [39]. Interestingly, the results suggest that NME5 may also regulate mitochondrial organization, although only NME3, NME4, and NME6 are recognized as mitochondrial-associated proteins in the NME family [40]. The loss of mitochondrial fluorescence in sperm from a patient harboring the *NME5* variant highlights its role in maintaining mitochondrial integrity, potentially linking it to energy metabolism and sperm motility [39].

NME5 has also been identified as a mediator of innate resistance to gemcitabine in pancreatic cancer cells, where its expression is controlled by the Sp1 transcription factor [41]. Furthermore, a study by Goc et al. showed that treatment of the human prostate cancer cell line PC3 with simvastatin resulted in reduced NME5 protein levels [42].

Comprehensive proteomic analysis of isolated human ciliary axonemes revealed that NME5 is also present in somatic airway epithelial cells, along with NME7 and NME9 [43]. In the Alaskan Malamute dog, a frameshift mutation in *NME5* results in primary ciliary dyskinesia (PCD), a disease characterized by chronic respiratory infection and impaired fertility. Numerous ultrastructural ciliary defects were noted, including abnormal numbers of microtubules and loss or shortening of dynein arms [44]. In 2020, a disease-causing mutation in NME5 was reported for the first time in a human patient with PCD. Functional studies (morpholino mediated knockdown of the ortholog gene in zebrafish embryos) confirmed defects in motile cilia, linking NME5 to ciliary structure, specifically radial spoke/central pair microtubule integrity. This established *NME5* as a true PCD-causing gene in humans [45,46].

Comprehensive analyses of the *NME* family in all six major eukaryotic supergroups (Opisthokonta, Amoebozoa, Plantae, Excavata, Chromalveolata, and Rhizaria) showed that NME5- and NME7-like proteins were already present in the ancestor of all eukaryotes and resembled the red alga *Chondrus crispus* NME5 (NME5-likeCc) [47]. Perina and coworkers revealed that the ancestral type of the NME5-like protein, in contrast to the human homolog, was a fully functional multimeric NDP kinase with DNA-binding capacity. The specific activities of the NME5-likeCc kinase were similar to and within the previously reported range for the human NME1/2 protein. In human, Yoon and colleagues showed that NME5, along with NME1, NME7, and NME8, exhibits 3′ → 5′ exonuclease activity but could not demonstrate the classical nucleoside diphosphate kinase activity [48]. However, it is possible that over evolutionary time, the NME5 lineage in higher eukaryotes lost the canonical NDPK activity, implying functional divergence [47].

Vertebrate NME5 contains a C-terminal Dpy-30 domain whose function is generally not well-understood. In Chlamydomonas reinhardtii, this domain, along with its flanking sequences, mediates the assembly and regulation of flagellar radial spoke complexes [49]. In Caenorhabditis elegans, the 123-amino-acid DPY-30 protein is essential for dosage compensation during early embryogenesis, and in males, it is also required for several developmental processes including coordinated movement, normal body size, proper tail morphology, and mating behavior [50]. In mammals, Dpy-30 participates in histone H3K4 methylation and plays a key role in specifying cell fate in embryonic stem cells [51].

**NME6**

The evolutionary paths of *NME5* and *NME6* are interconnected, as *NME6* arose from the duplication of an *NME5-like* gene early in the evolution of eukaryotes [52]. The human *NME6* gene and protein were first described in 1999 by two independent laboratories [53,54]. Both positioned the gene on chromosome 3p21.3 and described the corresponding protein as either 186 or 194 amino acids in length. The consensus NME6 protein, consisting of 186aa, has a molecular weight of 21.142 kDa and an isoelectric point of 8.5. Notably, NME6 differs from NME1 by a three-residue insertion within the Kpn loop and a 31-residue extension at the C terminus, while all residues of the catalytic pocket necessary for enzymatic activity are conserved [1,53].

Early immunofluorescence studies identified the proteins’ localization to mitochondria [54], which was further supported by large-scale proteomic analyses [55,56,57]. Our team confirmed the mitochondrial localization in live human cells using GFP reporters, and further refined its association to the mitochondrial inner membrane and matrix through mitochondrial purification and subfractionation [30].

The effect of *NME6* overexpression or silencing at the organism level remains unclear. In mice, the homozygous knockout (KO) of *NME6* is strongly correlated with embryonic lethality at early stage, while heterozygous knockout leads to neural development deficiencies [58]. NME6 has also been proposed to regulate inflammation in mouse models [59]. In human, *NME6* is overexpressed in gastric and colon cancer [60,61], and its overexpression correlates with poorer outcomes in lung adenocarcinoma [62]. Similarly, high expression of *NME6* has been reported in hepatocellular carcinoma and is associated with poor patient survival [63]. Conversely, low *NME6* expression has been identified as a marker of poor prognosis in late-stage colorectal cancers [64], while overexpression has been speculated to act as a protective factor in ovarian cancer [65]. These divergent observations indicate that the clinical significance of *NME6* is not uniform across different tumor types and point to its context-dependent role. Recently, a strong decrease in NME6 expression was reported in neutrophilic asthma [66], while silencing of *NME6* and *NME7* led to impaired embryonic stem cell renewal [67]. Noteworthy, in 2026, *NME6* expression was positively associated with higher blood pressure [68].

Data mining of mass spectrometry results revealed a consistent and strong interactor of NME6: RCC1L [56,69,70,71,72]. This interaction was confirmed by immunoprecipitation and the proximity ligation assay [30]. RCC1L is localized in the mitochondrial matrix [73,74], and is found to be essential for oxidative phosphorylation [69]. The protein is part of the pseudouridine synthase module, which is responsible for the maturation of specific mitochondrial RNAs (mt-RNA), an essential step in the production of mitochondria-encoded proteins, which are all key elements of the respiratory chain complexes [73,75]. Through its involvement in mt-rRNA maturation and interaction with mitoribosomal proteins, RCC1L is strongly associated with mitoribosome biogenesis [69,76]. As for NME6, homozygous *RCC1L* knockout in mice is lethal at early embryonic stages, while heterozygous knockout is associated with mitochondrial dysfunctions [77]. Expression of both proteins is closely connected, since the depletion of *RCC1L* leads to a strong decrease in NME6 protein [78], and vice versa [79]. Notably, a similar positive correlation was also observed in our laboratory, where *RCC1L* tends to be overexpressed in *NME6*-Knock-in cells (NME6-KI) (unpublished data).

The enzymatic NDPK activity of NME6 has been debated since the first description of the protein [48,54]. The classical chemical reaction, as described for all Group I proteins, involves the multimerization of NMEs as an obligatory step for catalytic site stabilization [17,80]. Our team demonstrated that NME6 was unable to form such oligomers, either isolated in vitro or in a cellular context, and therefore no NDPK activity was measured on the purified NME6 proteins [30]. However, a major breakthrough by Kramer et al. in 2023 demonstrated that the interaction between RCC1L and NME6 is sufficient to stabilize the latter protein and allow the NDPK reaction to occur [79]. Hence, this alternative stabilization mechanism might extend to other Group II NME family members, potentially revealing NDPK activity that has remained hidden to date. Nevertheless, this observation should be interpreted with caution, as it is currently based on a limited number of publications and experimental systems, and it remains unclear whether it reflects a broader principle applicable to other Group II NME proteins. Nevertheless, this discovery highlights that NME6, together with RCC1L, can indeed locally fuel the mitochondrial matrix with specific NTPs that are important for mitochondrial functions.

In tumor cell lines, overexpression or silencing of *NME6* has little to no effect on cell cycle progression or apoptosis. However, NME6-KI cells show a strong reduction in migration potential in the wound healing assay [81]. In contrast, NME6-KO cells display a decrease in cell proliferation [78,79]. Both positive and negative imbalances of *NME6* expression lead to a decrease in maximal respiration rate, correlated with dysregulated abundance of OXPHOS subunits [30,78,79]. Kramer et al. elegantly demonstrated that these effects were not rescued by re-expression of an enzymatically-dead mutant of NME6, providing evidence that the NDPK activity of NME6 is important for the biogenesis and maintenance of OXPHOS complexes [79]. In NME6-KO mitochondria, it was reported that there was an accumulation of pyrimidine mono- and diphosphate nucleotides (CMP, CDP, UMP, UDP), while only (d)CTP was strongly depleted [78,79].

As efficiently summarized by Wanrooij and MacVicar [82,83], NME6 appears highly important for the pyrimidine ribonucleotide salvage pathway within mitochondria, converting rCDP to rCTP and rUDP to rUTP, thus particularly supplying mt-RNA synthesis with rCTP and rUTP. On the other hand, the mtDNA replication is largely unaffected by NME6, due to other dNTP import pathways via specific transporters [78,79]. Therefore, at the molecular level, the loss of NME6 leads to a decrease in mt-RNA, which slows down mitochondrial translation and consequently depletes OXPHOS complexes of essential mitochondria-encoded proteins. At the mitochondrial level, this results in a decrease in maximal respiration rate, lowering ATP production via the OXPHOS pathway, and likely increasing mitochondrial stress. At the cellular level, this could potentially explain the observed decrease in cell proliferation.

**NME7**

The human *NME7* gene, also known as *nm23-H7* or *NDPK7*, is located on chromosome 1 at cytogenetic band 1q24.2. Expression profiling shows that NME7 is most abundant in the testis, while substantial expression is also detected in the ovary, brain, liver, heart, small intestine, and spleen [1]. In mice, *NME7* expression is particularly enriched in tissues containing motile cilia and in sperm [84]. NME7 was originally identified as a vital component of the γ-tubulin ring complex (γTuRC), a crucial protein structure for the nucleation of new microtubules [85,86]. In association with γTuRC, NME7 localizes to centrosomes throughout the cell cycle, including mitotic spindle poles during metaphase and basal bodies during ciliogenesis [87]. Although it does not appear to regulate γTuRC assembly or centrosomal targeting, NME7 is essential for efficient centrosome-dependent microtubule nucleation [86].

**Figure 1:**
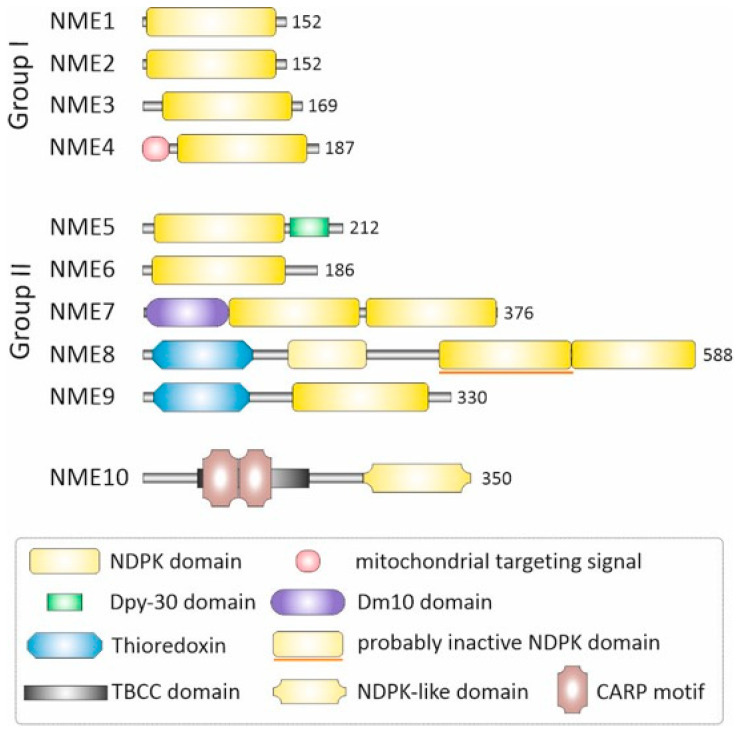
Schematic domain architecture of the NME protein family. Numbers indicate protein lengths in amino acids. Protein domains are represented by colored boxes, and each protein has been searched against the SMART/Pfam databases. Domain annotations and abbreviations were obtained from the SMART and Pfam databases.

In structural terms, NME7 differs from canonical NME family members by combining an N-terminal DM10 domain with two tandem NDPK-like domains, designated as domain A and domain B (Figure 1). The DM10 region is an unusual module of approximately 100 amino acids whose precise biochemical role remains incompletely understood [88]. Domain A retains autophosphorylation capacity on the catalytic histidine and is therefore considered as the catalytically competent domain, whereas domain B is catalytically inactive in this context [86]. Mutational analyses support this asymmetry, showing that substitutions in domain A abolish autophosphorylation, while analogous substitutions in domain B do not. However, domain B remains indispensable for NME7 function, as Arg-322 within this region is required for binding to the γ-tubulin ring complex [86].

Recent advances in cryo-electron microscopy have substantially expanded our understanding of NME7 at the ultrastructural level. High-resolution studies of the doublet microtubules (DMTs) in cilia and flagella—from the algal ortholog FAP67 [89], to sea urchin [90], and various mammalian systems including humans [90,91,92,93,94,95,96,97]—have consistently positioned NME7 inside tubule A of the DMT. In this configuration, domain A faces the solvent, whereas domain B interacts with the inner surface of tubule A. The striking conservation of this arrangement across respiratory cilia, fallopian tube cilia, sperm flagella, and motile algal flagella suggests that the ciliary function of NME7 arose early in evolution and has remained highly conserved.

The biological importance of NME7 is further demonstrated by the phenotypes associated with its loss. NME7 deficiency causes a mild accumulation of cells in the G1 phase of the cell cycle and increases the frequency of chromosome segregation errors [98], indicating that its functions may extend beyond ciliary organization to broader aspects of cell-cycle regulation and genome stability. In parallel, NME7 deficiency impairs ciliary assembly, increases sensitivity to the microtubule-disrupting agent nocodazole, and compromises Hedgehog signaling [87]. In vivo studies provide strong support for its physiological relevance in ciliary function and developmental patterning. In mice, NME7 knockout is associated with ciliary dyskinesia, *situs inversus totalis*, and hydrocephalus [99,100]. In humans, an in-frame deletion of 34 amino acids within domain B has been described in patients with *situs inversus totalis* [84], while homozygous deletion of NME7 in rats is semi-lethal and causes symptoms of primary ciliary dyskinesia, postnatal growth retardation, and sterility [101]. Collectively, these findings establish NME7 as an important regulator of ciliary integrity and left–right body asymmetry.

A particularly unusual aspect of NME7 biology is the NME7AB variant, a truncated recombinant form of human NME7 described as a regulator of embryonic stem cells. In this context, NME7AB has been reported to act as a primitive growth factor capable of maintaining the naïve state of human pluripotent stem cells in the absence of serum, FGF2, or other exogenous growth factors [102]. More broadly, both NME7 and NME6 have been implicated in regulating key pluripotency factors, including Oct4, Nanog, Sox2, and Klf4, while their depletion promotes spontaneous differentiation and reduces embryoid body formation [67]. These observations suggest a possible role for NME7 not only in differentiated ciliated tissues, but also in maintaining stem cell identity.

Beyond development and stem cell biology, NME7 has also been linked to metabolic phenotypes. A population-based study involving 1262 individuals with varying degrees of glucose tolerance identified five NME7 polymorphisms (rs4656659, rs2157597, rs10732287, rs4264046, and rs10800438) associated with biphasic glycemic curves, suggesting a metabolically protective effect [103]. Although the mechanistic basis of this association remains unclear, these findings raise the possibility that NME7 may influence systemic metabolic regulation in addition to its well-established cellular roles.

Accumulating evidence suggests that NME7 may also participate in tumor-related processes. Several studies indicate that NME7 can act as an oncogenic factor in specific contexts, most notably in hepatocellular carcinoma (HCC), where it has been linked to activation of the Wnt/β-catenin signaling pathway. In an HCC mouse model, *NME7* overexpression promoted tumorigenesis in cooperation with c-Myc, whereas *NME7* knockdown suppressed tumor growth [104]. In addition, the exposure of cancer cells to the NME7AB variant has been reported to increase the expression of metastatic markers and enhance tumor growth, while treatment with an anti-NME7AB antibody markedly reduced or abolished metastasis [105]. While these findings support a pro-tumorigenic role for NME7 in this setting, it remains uncertain whether these signaling effects are a feature of NME7 biology or a more restricted function in specific tumor contexts.

**NME8 and NME9**

Like other proteins of NME Group II, NME8 and NME9 have been poorly investigated to date. *NME8*, an ancient gene considered a metazoan innovation, is located on human chromosome 7p14.1. *NME9*, located on human chromosome 3q22.3, likely originated from an incomplete duplication of the *NME8* gene [25,33]. Both are associated with ciliary structures and are predominantly found in tissues containing ciliated cells. *NME8* was first characterized as *Sptrx-2* (spermatid-specific thioredoxin-2), as it was found to be expressed in human testicular germ cells and contains an N-terminal thioredoxin domain [106]. It has also been referred to as TXNDC3 (thioredoxin domain-containing protein 3) [107]; however, the presence of three NDPK domains following the N-terminal thioredoxin domain places this protein within the NME family, and it is therefore designated as NME8. Human NME9 was also initially characterized as a novel member of the thioredoxin family and named TXNDC6 (thioredoxin domain-containing 6) or TXL2 (thioredoxin-like 2), and was shown to be associated with microtubular structures such as the cilia of the lung airway epithelium, as well as the manchette and axoneme of spermatids [108]. Like NME8, NME9 possesses an N-terminal thioredoxin domain but contains only one NDPK domain. It seems that neither NME8 nor NME9 display detectable thioredoxin or NDPK activity [48,106,108].

The functions of NME8 are primarily associated with microtubule dynamics and cytoskeletal organization. It is highly expressed in testicular tissue where it likely contributes to the axonemal structure and function of sperm flagella [106]. Alterations in the *NME8* gene that change the ratio of its two transcripts are implicated in primary ciliary dyskinesia, a disorder of axonemal structure characterized by respiratory infections and male infertility [107]. NME8 is also emerging as a mediator of cisplatin-induced reproductive toxicity following cancer treatment. In mice, NME8 is involved in the response to cisplatin. Its deficiency (due to deletion of exons 6–7) exacerbates cisplatin-induced oxidative stress, further impairs antioxidant defense mechanisms and autophagy, and increases DNA damage in the sperm and testes, resulting in reduced sperm production and motility under cisplatin exposure. These findings suggest that NME8 plays a protective role against cisplatin-induced damage in the testes by maintaining redox balance during oxidative stress [109].

Consistent with a redox-protective role, *NME8* expression has been associated with protection against paternal age-related increases in oxidative stress [110]. Although both NME8 and NME9 contain a thioredoxin domain, neither displays intrinsic thioredoxin-reducing activity [106,108]. However, deletion of this domain in sperm increases susceptibility to oxidative stress, suggesting that NME8 functions as a redox regulator [110].

According to Yoon and coworkers, NME8 exhibits 3′–5′ exonuclease activity [48], similar to some other NME proteins, including NME1, NME5, and NME7, with a preference for single-stranded DNA. This activity suggests that reduced *NME8* expression may contribute to genetic instability and cancer progression [111]. However, despite these disease associations, much of the current evidence linking NME8 to cancer and other pathologies comes from broad genetic, transcriptomic, and biomarker-based studies, while the underlying molecular mechanisms remain poorly characterized.

In the context of cancer, the current literature on NME8 is more informative regarding associations than mechanisms. *NME8* overexpression has been observed in renal cancer and is strongly correlated with poor prognosis. In vitro, depletion of NME8 suppresses the proliferation, migratory potential, and invasion of renal cancer cells, and promotes apoptosis [112]. The ability of NME8 to promote metastasis is mediated through the Janus kinase (JAK)/signal transducer and activator of transcription (STAT) pathway. Furthermore, NME8 is involved in the regulation of the tumor microenvironment and its expression correlates with immune cell infiltration. In chronic myeloid leukemia, *NME8* is also upregulated and identified as a hub gene that negatively correlates with the activation of tumor-associated pathways [113]. Similarly, it is identified as a biomarker in diffuse large B-cell lymphoma, the most common type of non-Hodgkin lymphoma, and its expression was reduced in patients with systemic and testicular lymphoma [114,115]. Furthermore, *NME8* has been suggested to be a candidate predisposition gene in BRCA1/BRCA2-negative familial breast cancer [116]. The NME8 locus has also been identified as a disease-associated genomic region in periodontitis [117], osteoporosis [118], and knee osteoarthritis [119]. Polymorphisms in the *NME8* gene have also been associated with increased risk of Alzheimer’s disease [120,121], particularly late-onset forms [122,123], potentially through correlation with increased PTK2B expression and MAPK pathway regulation [124]. *NME8* polymorphisms also contribute to idiopathic normal pressure hydrocephalus, a progressive brain disease that usually appears in comorbidity with Alzheimer’s disease [125]. Taken together, these observations indicate that NME8 is associated with diverse pathological processes, but at this stage, there is no unified mechanistic framework. Therefore, it remains unclear whether NME8 acts directly within oncogenic signaling pathways or whether many of its disease associations reflect roles in ciliary organization, microtubule-associated architecture, redox control, or genome maintenance.

*NME9* is expressed in the testis and in ciliated cells of the lung where it binds microtubules, consistent with its structural similarity to *NME8*. Interestingly, NME9 has been found in multiple cilia-containing areas of the respiratory tract, as well as in the female reproductive tract [126]. As for the male reproductive tract, NME9 can be detected only in the early stages of spermatogenesis. Indeed, NME9 is present in motility-deficient spermatids, but is absent from fully developed and motile spermatozoid, indicating a role in flagella formation [97,108]. Although functional characterization of NME9 is limited, its expression in ciliated tissues suggests a role in ciliary physiology. In cancer, cytoplasmic expression of *NME9* was associated with a negative outcome in systemic diffuse large B-cell lymphoma, indicating its potential role in tumor biology; however, the available evidence remains largely correlative, and the underlying mechanism is still unknown [115].

## 2. Conclusions

Group II NME proteins differ substantially from one another and from their orthologs in other species, especially when contrasted with the more conserved NME1–4 subgroup. Despite this diversity, several unifying features emerge. NME5, NME7, NME8, and NME9 consistently localize to cilia and flagella—whether during spermatogenesis, within the respiratory epithelium, or in the fallopian tube. Although cilia and flagella vary in number and length, they share a common structural organization characterized by the 9 + 2 axonemal arrangement of microtubules. Disruption or loss of NME proteins associated with these structures, or with microtubules themselves (as in the case of NME7), is linked to ciliopathies such as primary ciliary dyskinesia (NME5, NME8, NME9) and *situs inversus totalis* (NME7) (Table 1).

Microtubules elongate by incorporating GTP bound tubulin dimers at their plus ends, and the GTP cap is essential for stabilizing the growing tip [127]. This raises the possibility that a locally maintained pool of GTP is required within cilia and flagella. In this context, it is reasonable to speculate that NME proteins positioned within these structures may provide GTP in a non-canonical manner, potentially independent of the classical hexameric organization previously thought to be essential for NDPK activity. Indeed, Group I NME members such as NME1 [3] or NME4 [128] have been implicated in similar processes, including local GTP supply for dynamin related GTPases such as OPA1. However, this idea currently remains more of a working hypothesis than an experimentally established mechanism.

For decades, Group II NME members were considered catalytically inactive with respect to NDPK function, with the possible exception of NME6. However, accumulating evidence challenges this long-standing assumption. Notably, Perina et al. demonstrated that NME5 in algae retains measurable NDPK activity, providing early evidence that catalytic potential may persist in specific evolutionary or structural contexts [47].

Moreover, the dogma that hexamerization is necessary for NDPK activity has been challenged by recent findings. It was demonstrated that NME6 cannot assemble into higher-order oligomers, either in vitro or in the cellular context [30]. While monomeric recombinant NME6 did not show NDPK activity when isolated in vitro, two independent studies have shown that NME6 can exhibit NDPK activity in the presence of the mitochondrial protein RCC1L. The interaction between NME6 and RCC1L was sufficient to stabilize the catalytic site of NME6 and enable enzymatic activity [77,78]. These findings represent a significant shift in our understanding of NME biology and call into question the 30-year-old paradigm that NDPK activity is restricted to hexameric NME assemblies. Nevertheless, they should be interpreted with caution, as the currently available evidence remains limited. More generally, these results raise the possibility that noncanonical mechanisms—such as stabilization by partner proteins or physical constraints imposed by specialized subcellular environments, for example, NME7 positioned within the microtubule lumen—could permit catalytic activity in additional Group II NME proteins. However, such an extension remains hypothetical and is not yet supported by direct experimental evidence.

Beyond their potential enzymatic roles, Group II NME members participate in a broader spectrum of cellular processes. NME5 and NME8 have been implicated in protection against reactive oxygen species, while NME6 and NME7 seem to be essential for embryonic stem cell renewal. Moreover, NME6–NME9 have all been associated with cancer-related phenotypes, while such evidence is currently lacking for NME5. Important aspects of this subgroup nevertheless remain unresolved, including the biological significance of the thioredoxin domains in NME8 and NME9. Overall, the emerging biology of NME6, which reframes the previous model of NDPK activity, suggests that Group II NME proteins should not be viewed simply as catalytically inactive variants of canonical NDPKs, but rather as structurally and functionally diverse proteins whose activities may depend strongly on cellular context, interaction partners, and subcellular localization.

## Figures and Tables

**Table 1:** Group II NME proteins—comprehensive feature summary (NME5–NME9).

Protein	Synonyms	Gene Locus (Chr:Pos)	Proven Enzymatic Activity	Subcellular Location	Cilia/Axoneme/ Microtubule Role	Associated Diseases	Cancer Associations	Other Key Functions
**NME5**	NM23-H5; RSPH23; NDK5 UniProt P56597 OMIM gene 603575	5q31.2	• DISPLAY 3′ → 5′ exonuclease activity [48] • LACK NDPK activity [48]	• Sperm flagellum [34,35] • Respiratory Cilia [46] • RS1 stalk/head-neck complex of axonemal radial spoke [35,92,97]	• Lost of NME5 = Ciliary defects [35,39,44,46]	• Primary ciliary dyskinesia (PCD) [44,46] • Male infertility (connected to PCD) [35,39] • Acephalic Spermatozoa syndrome [39] • Respiratory tract Infection (connected to PCD) [44,46]	• NO clear link	• ROS protection during spermatogenesis (GPX5 pathway) [37]
**NME6**	NM23-H6; Nm23-M6; NDK6; IPIA-ALPHA UniProt O75414 OMIM gene 608294	3p21.3	• LACK NDPK activity as monomer [30] • GAINS NDPK activity upon oligomerization with RCC1L [78]	• Mitochondrial matrix/inner membrane (MIM) [30] • Co-localizes with mt-DNA nucleoids and mt-RNA granules [78]	• No cilia/axoneme role reported • Sole Group II NME exclusively mitochondrial [40]	• NME6-KO = Early lethality in mice [58] • NME6 KO → OXPHOS destabilization, ETC disruption, mt-RNA depletion [30,78] • Inflammation [59]	• NME6 High expression in colon cancer, lung cancer and hepatic cancer = Poor prognosis [60,61,62,63] • NME6 low expression in colon cancer = Poor prognosis [63] • NME6 high expression in Ovarian cancer = Better prognosis [65]	• Essential for ESC renewal [67]
**NME7**	NM23-H7; NDK7; CFAP67; MN23H7 UniProt Q9Y5B8 OMIM gene 613465	1q24.2	• DISPLAY Protein kinase activity: phosphorylates GSK3β-Ser9 (Wnt activation) [104] • DISPLAY 3′ → 5′ exonuclease activity [48] • LACK NDPK activity [25,48]	• Cilia (Respiratory tract, fallopian tube, sperm flagella) [90,91,92,93,96] • Within the doublet of Microtubule [90,91,92,93,96] • Centrosome (γ-TuRC component) [85,87]	• γ-TuRC component [84] • Required for primary cilium assembly and ciliary Microtubules stability [87]	• Situs inversus totalis [84,99] • Glucose metabolism [103] • Semi-lethal PCD in Nme7 KO rats: situs inversus, immotile cilia, sterility, respiratory tract infection [101]	• Oncogenic driver in Hepatocellular carcinoma = Poor prognosis [104]	• Essential for ESC renewal [67]
**NME8**	TXNDC3; SPTRX2; NM23-H8; CILD6; DNAI8; HEL-S-99 UniProt Q8N427 OMIM gene 607421	7p14.1	• LACK NDPK activity [48,106] • DISPLAY Autophosphorylation [48] • LACK thioredoxin reductase activity [106] • DISPLAY 3′ → 5′ exonuclease [48]	• Cilia (Respiratory tract, sperm flagella) [106,107]	• Outer dynein arm component [107]	• Primary ciliary dyskinesia (PCD) [107] • Male infertility (connected to PCD) [107] • Respiratory tract Infection (connected to PCD) • Risk of Alzheimer’s disease [120,121]	• NME8 High expression in renal cancer, Chronic myeloid leukemia = Poor prognosis [112,113] • NME8 low expression in lymphoma [114,115]	• ROS protection: Maintain redox balance during oxidative stress [109,110]
**NME9**	TXNDC6; TXL-2; TXL2; NM23-H9; NXL2 UniProt Q86XW9 OMIM gene 618584	3q22.3	• LACK thioredoxin reductase activity [108] • LACK NDPK activity [108]	• Ciliae (Respiratory tract, nasopharynx, bronchus, fallopian tube, endometrium, cervix) [108,126] • PRESENCE of NME9 in Spermatid (early stage of spermatozoid, without flagella) [108] • ABSENCE of NME9 in sperm-motile Flagella [97]	• Potential role in Sperm Flagella FORMATION ONLY [97,108]	• CANDIDATE for Primary ciliary dyskinesia (PCD) [107]	• NME9 cytoplasmic expression in lymphoma = Poor prognosis [115]	• ROS protection: Maintain redox balance during oxidative stress [109,110]

Abbreviations: NDPK—nucleoside diphosphate kinase; PCD—primary ciliary dyskinesia; ROS—reactive oxygen species; RS1—radial spoke 1; γ-TuRC—γ-tubulin ring complex; ESC—embryonic stem cell. ETC—electron transport chain; mt-DNA—mitochondrial DNA; mt-RNA—mitochondrial RNA; OXPHOS—oxidative phosphorylation. Note: NME6 is the only Group II NME with no reported cilia/axonemal localization.

## Data Availability

No new data were created or analyzed in this study. Data sharing is not applicable to this article.

## Abbreviations

The following abbreviations are used in this manuscript:

ASS	Acephalic spermatozoa syndrome
ATP	Adenosine triphosphate
CDP	Cytidine diphosphate
CMP	Cytidine monophosphate
CTP	Cytidine triphosphate
DMT	Doublet microtubules
GFP	Green fluorescent protein
GPX-5	Glutathione peroxidase 5
GTP	Guanosine triphosphate
HCC	Hepatocellular carcinoma
JAK/STAT	Janus kinase/signal transducer and activator of transcription
KO	Knockout
mt-RNA	Mitochondrial RNA
NDP	Nucleoside diphosphate
NDPK	Nucleoside diphosphate kinase
NME	NME protein family
NME6-KI	NME6 knock-in cells
fNTP	Nucleoside triphosphate
PCD	Primary ciliary dyskinesia
RCC1L	Regulator of chromosome condensation 1-like protein
Sptrx-2	Spermatid-specific thioredoxin 2
TBCC	Tubulin-binding cofactor C
TXL2	Thioredoxin-like 2
TXNDC3	Thioredoxin domain-containing protein 3
TXNDC6	Thioredoxin domain-containing protein 6
UDP	Uridine diphosphate
UMP	Uridine monophosphate
UTP	Uridine triphosphate
γTuRC	γ-Tubulin ring complex
